# Validation and Functional Analysis of Reference and Tissue-Specific Genes in Adipose Tissue of Freshwater Drum, *Aplodinotus grunniens*, under Starvation and Hypothermia Stress

**DOI:** 10.3390/cells12091328

**Published:** 2023-05-06

**Authors:** Miaomiao Xue, Haibo Wen, Pao Xu, Jianxiang Chen, Qingyong Wang, Yongkai Tang, Xueyan Ma, Guohua Lv, Hongxia Li, Changyou Song

**Affiliations:** 1 Wuxi Fisheries College, Nanjing Agricultural University, Wuxi 214081, China; 2 Key Laboratory of Freshwater Fisheries and Germplasm Resources Utilization, Ministry of Agriculture and Rural Affairs, Freshwater Fisheries Research Center, Chinese Academy of Fishery Sciences, Wuxi 214081, China

**Keywords:** *Aplodinotus grunniens*, tissue-specific genes, feed-restriction, cold stress, abdominal fat

## Abstract

Adipose tissue is critical to the growth, development, and physiological health of animals. Reference genes play an essential role in normalizing the expression of mRNAs. Tissue-specific genes are preferred for their function and expression in specific tissues or cell types. Identification of these genes contributes to understanding the tissue–gene relationship and the etiology and discovery of new tissue-specific targets. Therefore, reference genes and tissue-specific genes in the adipose tissue of *Aplodinotus grunniens* were identified to explore their function under exogenous starvation (1 d, 2 w, 6 w) and hypothermic stress (18 °C and 10 °C for 2 d and 8 d) in this study. Results suggest that 60SRP was the most stable reference gene in adipose tissue. Meanwhile, eight genes were validated as tissue-specific candidates from the high-throughput sequencing database, while seven of them (ADM2, β_2_GP1, CAMK1G, CIDE3, FAM213A, HSL, KRT222, and NCEH1) were confirmed in adipose tissue. Additionally, these seven tissue-specific genes were active in response to starvation and hypothermic stress in a time- or temperature-dependent manner. These results demonstrate that adipose-specific genes can be identified using stable internal reference genes, thereby identifying specific important functions under starvation and hypothermic stress, which provides tissue-specific targets for adipose regulation in *A. grunniens*.

## 1. Introduction

Adipose tissue of fish is distributed throughout the body (liver, brain, subcutaneous, abdominal cavity, and red and white muscles). Different parts of adipose tissue serve different functions, such as that in the subcutaneous and peri-insular areas, which influences the carcass and fillet yields, and in the muscle depots, which regulates the organoleptic quality of the meat [1]. Adipose tissue regulates systemic metabolic homeostasis through its profound effects on energy storage, endocrine function, and adaptive thermogenesis. When the feed intake exceeds their expenditure, excessive energy will converse into triglycerides and store in the body as fatty acids, which can in turn serve as an energy supply under feed restriction [2]. As an endocrine organ, adipose tissue functions importantly in nutritional, neurological, hormonal, and metabolic regulation by synthesizing bioactive compounds and regulating metabolic homeostasis [3], such as triglyceride synthesis and storage, adipokine secretion, and overall energy balance [4,5].

Generally, fats in the body are deposited in adipose tissue. At present, most economically important fish are fed with compound diets, which are generally characterized by energy surpluses and nutrient imbalances. Similarly with mammals, excessive fat accumulation in fish represents a disturbed metabolic condition that not only damages the health of the fish but also reduces the quality of the product [6,7]. Meanwhile, excessive lipid deposition also negatively alters the morphology of tissues or cells, resulting in a variety of negative effects on fish health [8,9]. Therefore, reducing excessive lipid deposition contributes to improving the health and quality of aquatic products. Feed restriction has been shown to reduce abdominal fat accumulation [10,11]. During this process, exogenous food intake was restricted, while the endogenous substances were active to generate energy for the maintenance of normal physiological activity, such as carbohydrates, lipids, and proteins [12,13,14]. Research in *Danio rerio* revealed that carbohydrate is principally used for energy supply during the early stage of starvation (within 24 h), while fat is mobilized for energy supply during 2~4 d of starvation. However, protein is barely expended for energy supply during short-term starvation [15]. Therefore, short-term feed restriction is an effective approach to revealing the lipid metabolism of animals.

The growth, physiology, and reproduction of fish are greatly affected by the ambient temperature [16]. When temperature changes exceed the adaptive range of aquatic animals, hypo- or hyperthermic stress can cause metabolic disorders, tissue damage, and even death [17,18]. Under hypothermia, aquatic animals unconsciously consume more endogenous energy substances to increase energy production. Several studies indicate that lipid metabolism functions importantly in hypothermia resistance. Specifically, to cope with hypothermia, fish will alter their lipid metabolism. In order to compensate for the effects on enzyme function, fish will increase their membrane fluidity in response to the drop in ambient temperature [19,20,21,22]. Changes in phospholipid composition and cholesterol content [23] are effective ways to accomplish this. In response to hypothermic stress, fish can change the fatty acid composition of various tissue cell membranes by controlling the expression of enzymes and genes implicated in fatty acid metabolism [24]. Therefore, lipid metabolism in adipose tissue can alleviate the side effects of hypothermic stress and protect the health of the body.

Tissue-specific genes are a class of genes with specific functions in particular tissues or cells [25]. Identifying the function of these genes provides a better understanding of tissue genetic relationships, which can contribute to the discovery of new tissue-specific targets. In aquatic animals, tissue-specific genes have also been identified in some species, such as ovarian development-related genes in *Macrobrachium nipponense* [26], growth and feeding-related genes in *Aristichthys nobilis* [27] and immune-related genes in *Cyprinus carpio* [28]. In this paper, adipose-specific genes were identified, and the molecular regulation mechanisms in lipid metabolism were investigated in freshwater drums.

Freshwater drum (*Aplodinotus grunniens*) belongs to *Aplodinotus* and inhabits only freshwater during its lifetime [29]. In the aspect of edibility, freshwater drum features high edible proportions and delicious and nutritious flesh. With these prospects, we imported freshwater drum larvae and achieved a worldwide breakthrough in the advanced research of artificial breeding, feeding and domestication. In 2022 we achieved large-scale fry breeding, laying the groundwork for the industrialized development of freshwater drum culture and the breeding of new varieties [30,31,32,33]. However, the molecular mechanisms of adipose tissue in freshwater drums are hitherto unknown. Therefore, it is necessary to screen out the target molecules of adipose tissue, to understand the role of adipose tissue in freshwater drum development, and contribute to subsequent functional genomics.

Therefore, the above updated research progress inspired us to conduct this study to identify the stable internal reference genes, thereby validating the tissue-specific genes that function importantly in the adipose tissue of freshwater drum. Consecutively, we also validated whether these tissue-specific genes function importantly with starvation and hypothermic stress, which could induce metabolic and adaptive physiological dysfunction of adipose tissue. These results are novel in understanding the regulation mechanism of adipose tissue in freshwater drums. At the same time, these vital genes may also serve as candidate targets for starvation and hypothermia resistance, which are conducive to the sustainable development of freshwater drum.

## 2. Materials and Methods

### 2.1. Ethics Statement

This research has received approval from the Animal Care and Use Committee of Nanjing Agricultural University (Nanjing, China). All animal procedures were performed in accordance with the Guideline for the Care and Use of Laboratory Animals in China.

### 2.2. Experimental Animals and Experimental Design

The laboratory fish in the present study were the first-generation larvae of freshwater drums introduced from the United States. The experiment was carried out in an indoor temperature-adjustable circular aquaculture system (specifications for φ 820 mm × 700 mm, 300 L) of the Freshwater Fisheries Research Center, Chinese Academy of Fishery Sciences. Water was obtained from a deep well and aerated before use. Before the experiments, the fish were acclimated in a water tank at 25 °C for 14 days. The fish were fed with fresh bait twice daily (8:00 and 16:00, 3–5% weight).

For the starvation experiment, a one-factor, completely randomized experiment was designed. Four treatments were established, starvation for 0 d was set as the control, as followed by starvation for 1 d, 2 w, and 6 w. Two hundred and forty fish with an average body weight of 20.88 ± 2.75 g were divided into four groups (three tanks per group, 20 individuals per tank). During the experiment, all fish were not fed.

For the hypothermia experiment, a two-factor, completely randomized experiment was designed. Specifically, 25 °C was set as the control, 18 °C and 10 °C were set as hypothermia treatment for 2 d and 8 d, and six treatments were finally established (Con-2 d, LT18-2 d, LT10-2 d, Con-8 d, LT18-8 d, and LT10-8 d). One hundred and eighty fish were randomly divided into three groups (three tanks per group, 20 individuals per tank). The water temperature was decreased gradually from 25 °C to 18 °C and 10 °C in 15 h at a rate of 1 °C/h in the recirculating aquaculture system and maintained for eight days. During the hypothermia experiment, fish were fed as previously.

In this study, starvation and hypothermic stress experiments were performed as two independent experiments. Throughout all experiments, the water parameters were kept as follows: dissolved oxygen > 6 mg L^−1^, pH 7.2~7.8, NO_2_^−^ < 0.02 mg L^−1^, and NH_3_ < 0.05 mg L^−1^.

### 2.3. Sample Collection

The starvation experiment samples were collected at 0 d (control), 1 d, 2 w, and 6 w. Three fish were collected per tank at each time point for a total of nine biological replicates. The hypothermia experimental samples were collected at 2 d and 8 d at 25 °C (control), 18 °C, and 10 °C, respectively. At each temperature, three fish were collected from each replicate at each time point, for a total of nine biological replicates. In the hypothermic stress experiment, the fish were starved for 24 h to evacuate the alimentary tract contents before collecting samples. In the process of sampling, fish from each tank were randomly taken and anesthetized with MS-222 (100 mg/L), after which the abdominal fat (AF) was collected, immediately frozen in liquid nitrogen, and stored at −80 °C. Meanwhile, 15 tissues (including the brain (BR), head kidney (HK), trunk kidney (TK), liver (LI), foregut intestine (FI), mid-intestine (MI), hind intestine (HI), muscles (MU), abdominal fat (AF), gill (GI), spleen (SP), heart (HE), skin (SK), stomach (ST), and testis (TE)) from the fish reared at 25 °C were collected to conduct tissue-specific and internal reference gene analyses.

### 2.4. Extraction of Total RNA and Synthesis of cDNA

Total RNA was extracted from the tissues using TRIzol reagent (Invitrogen, Carlsbad, CA, USA). RNA quality and concentration were determined by A260/280 and an ultra-micro spectrophotometer, respectively. Subsequently, cDNA was synthesized with PrimeScript^TM^ RT Master Mix reverse transcription kit (Takara, Dalian, China). The above operations were carried out according to the instruction manual.

### 2.5. Screening of Reference and Tissue-Specific Genes

The candidate reference genes were selected from the published paper, including beta-actin (β-actin), elongation factor 1-alpha (EF1α), beta-2 microglobulin (B2M), 60S ribosomal protein (60SRP), glyceraldehyde-3-phosphate dehydrogenase (GAPDH), 18S Ribosomal RNA (18S), eukaryotic translation elongation factor1-beta (EEF1B), ribosomal protein L7 (RPl7), ribosomal protein S4 (RPS4), translocation protein SEC62 (SEC62), and ubiquitin-fold modifier 1 (ufm1). ΔCT, GeNorm, NormFinder, Bestkeeper, and RefFinder were used to assess the expression stability of candidate reference genes. Meanwhile, candidate tissue-specific genes were retrieved from the transcriptome database using 11 tissues from freshwater drums.

### 2.6. RT-PCR Analysis

Based on the mRNA sequences obtained from the transcriptome database, the primers were designed with Primer Premier 5.0. All the primers (shown in Table 1) were synthesized by Shanghai Generay Biotechnology, Co., Ltd., China. According to the manufacturer’s protocol, RT-PCR was performed with SYBR Green (Takara, Dalian, China) on Takara 800 Fast RT-PCR System.

### 2.7. Statistical Analysis

In the study, all data were calculated using SPSS software (version 26.0) and presented as mean ± standard error mean (SEM). The most stable internal reference genes were screened by ΔCT, GeNorm, NormFinder, Bestkeeper, and RefFinder. For RNA expression analysis, 2^−∆∆CT^ method was applied. For statistical difference evaluation, data was analyzed by one-way analysis of variance (ANOVA) followed by Duncan’s multiple range test when data was under normal distribution and homoscedasticity, or else using a nonparametric test (Kruskal–Wallis test). In the starvation experiment, Students’ *t*-test was used to analyze the difference between different starvation times. In the hypothermia experiment, two-way ANOVA was used to verify the interaction between time and temperature. To evaluate the impact of different temperatures and stress times on the expression of tissue specific genes, Student’s *t*-test and one-way ANOVA was applied, respectively. Pearson’s correlation analysis was used to assess the relevance of genes under starvation and hypothermic stress. In general, *p* < 0.05 was considered to be a significant difference or correlation, while *p* < 0.01 was regarded as an extremely significant difference or correlation.

## 3. Results

### 3.1. RNA Quality and Primer Amplification Assessment

**Figure 1:**
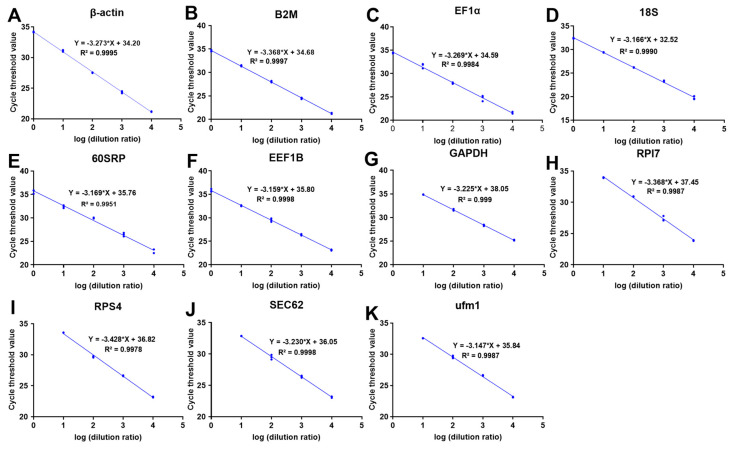
Standard curve of internal reference genes in *A. grunniens*. (**A**) beta−actin, β−actin; (**B**) beta−2 macroglobulin, B2M; (**C**) elongation factor 1−alpha, EF1α; (**D**) 18S Ribosomal RNA, 18S; (**E**) 60S ribosomal protein, 60SRP; (**F**) eukaryotic translation elongation factor1−beta, EEF1B; (**G**) glyceraldehyde−3−phosphate dehydrogenase, GAPDH; (**H**) ribosomal protein L7, RPl7; (**I**) ribosomal protein S4, RPS4; (**J**) translocation protein SEC62, SEC62; (**K**) ubiquitin−fold modifier 1, ufm1.

The A260/280 ratio was 1.98 ± 0.09, confirming that the RNA was pure and protein-free. Meanwhile, the RNA integrity number (RIN) was 9.7 ± 0.2, indicating that the RNA integrity was stabilized and reliable for RT-PCR analysis (Figure S1). Additionally, the amplification efficiency was between 95% and 108% (Table 1), and the coefficient of determination (R^2^) for each gene ranged from 0.9951 to 0.9998 (Figure 1A–K), which indicated that the primers were suitable for RT-PCR analysis.

### 3.2. Expression Ranges and Stability of Candidate Reference Genes

**Figure 2:**
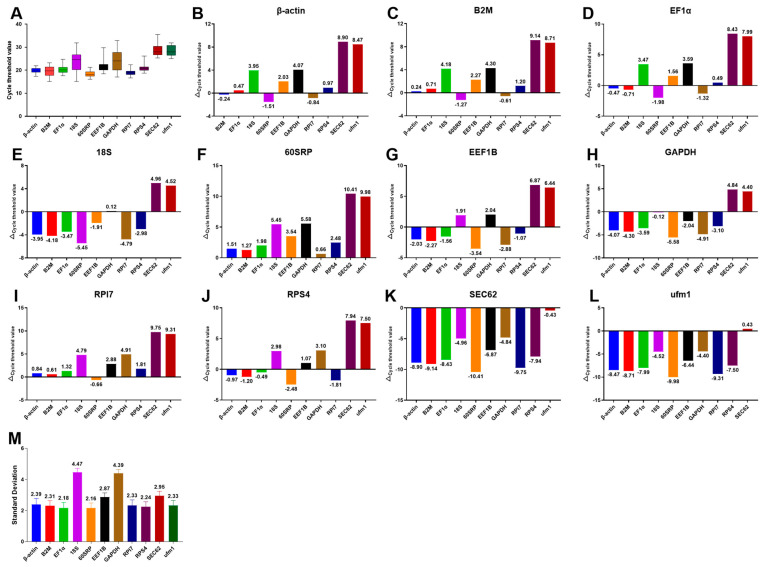
Expression ranges and stability of candidate reference genes in *A. grunniens*. (**A**) cycle threshold values for 11 candidate reference genes; (**B**–**L**) Δ cycle threshold analysis, (**B**–**L**) were controlled by β−actin, B2M, EF1α, 18S, 60SRP, EEF1B, GAPDH, RP17, RPS4, SEC62 and ufm1 respectively; (**M**) standard deviation of 11 genes. Box plot, represented by median, quartile, and CT value range.

Next, we determined the expression of candidate reference genes in terms of CT values by RT-PCR. The results show that the CT values of the 11 internal reference genes ranged from 15.05 to 35.5 (Figure 2A). However, the CT value of SEC62 reached outside the valid range of detection, implying low mRNA abundance in tissues, and indicating that it was unsuitable as a reference gene for freshwater drum.

**Figure 3:**
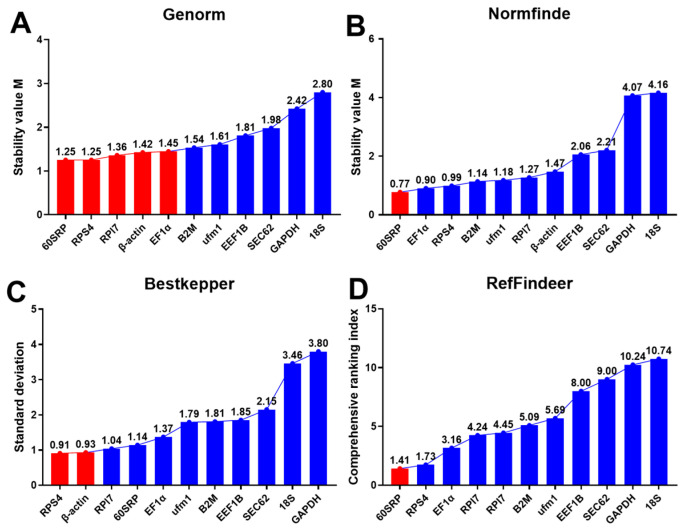
Expression stability analysis of candidate reference genes. (**A**) GeNorm analysis (M = 1.5 is critical value); (**B**) Normfinder analysis; (**C**) Bestkeeper analysis (SD < 1 is stable); (**D**) RefFinder analysis. Under different screening conditions, the red bars represent stably expressed genes and the blue bars represent unstable genes.

ΔCT analysis revealed that 60SRP was frequently associated with the least deviation, implying that it has low variability compared to the other ten genes (Figure 2B–M). GeNorm analysis demonstrated that 60SRP, RPS4, RPl7, β-actin, and EF1α exhibited high expression stability, as identified by the M value (M < 1.5, Figure 3A). NormFinder analysis (Figure 3B) confirmed that 60SRP had the highest expression stability among the 11 reference genes. Bestkeeper can calculate the standard deviation (SD) and coefficient of variation (CV). The SD indicated that only RPS4 and β-actin can be used as reference genes (SD < 1, Figure 3C), and the CV indicated that RPS4 had the smallest coefficient of variation and better stability (Table S1). RefFinder analysis showed that 60SRP was the most stable reference gene (Figure 3D).

### 3.3. Identification and Expression of Adipose Tissue-Specific Genes

**Figure 4:**
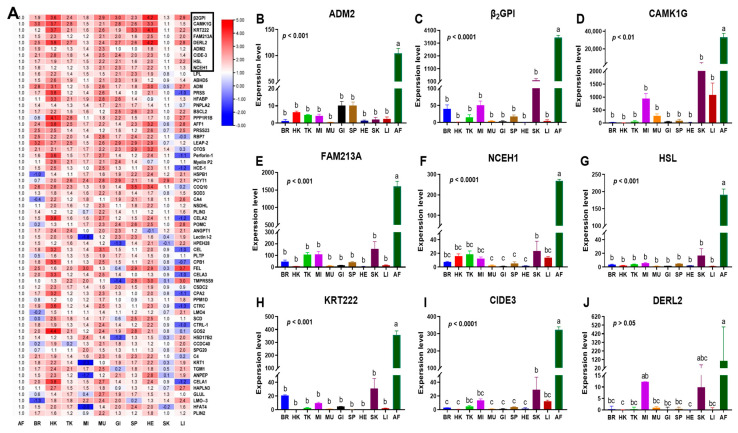
Identification of tissue−specific candidate genes in AF from RNA−seq. (**A**) Transcriptomic analysis heatmap of the fold change in the expression of AF and 10 other tissues in *A. grunniens*. The data in the heat map represents the log (fold change) value. (**B**–**J**) transcriptional expression of tissue−specific candidate genes in different tissues of *A. grunniens*, (**B**) adrenomedullin 2, ADM2; (**C**) beta−2−glycoprotein 1−like, β_2_GP1; (**D**) calcium/calmodulin-dependent protein kinase type 1G, CAMK1G; (**E**) cell death activator CIDE−3−like, CIDE3; (**F**) redox−regulatory protein FAM213A−like, FAM213A; (**G**) hormone−sensitive lipase, HSL; (**H**) keratin−like protein KRT222, KRT222; (**I**) neutral cholesterol ester hydrolase 1, NCEH1; (**J**) derlin−2−like, DERL2. Data were analyzed by one−way ANOVA. Different letters indicate marked differences between AF and the other 10 tissues (*p* < 0.05), results were indicated as mean ± SEM or medians ± interquartile range; *n* = 3.

Based on the above studies, tissue-specific genes were validated in freshwater drum adipose tissue using stable internal reference genes. According to the transcriptome database of 11 different tissues in freshwater drums, a total of 9 tissue-specific genes were selected (fold change > 10, *p* < 0.05; shown in Figure 4). Moreover, the expression of derlin-2-like (DERL2) in AF exhibited no significant difference with MI and SK (KW, *p* > 0.05), while the other candidate genes in AF exhibited a remarkable difference with all the other tissues (ANOVA, *p* < 0.01). Therefore, adrenomedullin 2 (ADM2), β2-glycoprotein I (β_2_GP1), calcium/calmodulin-dependent protein kinase type 1G (CAMK1G), keratin-like protein KRT222 (KRT222), cell death activator CIDE3-like (CIDE3), hormone-sensitive lipase (HSL), neutral cholesterol ester hydrolase 1 (NCEH1), and redox-regulatory protein (FAM213A) were identified as the candidate adipose tissue-specific genes.

### 3.4. Expression of Adipose Tissue-Specific Genes in Different Tissues

Next, we used RT-PCR to further verify whether the candidate genes were specifically expressed in adipose tissue. The amplification efficiency and coefficient of determination (R^2^) of the tissue-specific genes were determined. Results show that the amplification efficiencies were between 92% and 111% (Table 1), and the R^2^ for each gene ranged from 0.9541 to 0.9988 (Figure S2), indicating that the primers of tissue-specific genes were suitable for RT-PCR experiments.

**Figure 5:**
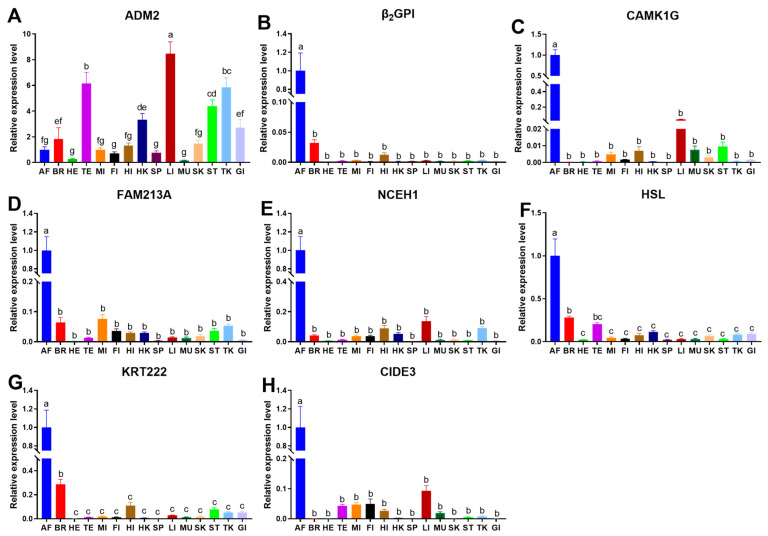
RT-PCR expression of tissue-specific candidate genes in different tissues of *A. grunniens*. (**A**), ADM2; (**B**), β_2_GP1; (**C**), CAMK1G; (**D**), CIDE3; (**E**), FAM213A; (**F**), HSL; (**G**), KRT222; (**H**), NCEH1. Data were analyzed by one-way ANOVA. Different letters indicate significant differences between AF and other tissues (*p* < 0.05), results are indicated as mean ± SEM; *n* = 9.

The expressions of β_2_GP1, CAMK1G, CIDE3, FAM213A, HSL, KRT222, and NCEH1 were significantly more abundant in AF than in other tissues (ANOVA, *p* < 0.05), while ADM2 exhibited no remarkable difference in BR, HE, MI, FI, HI, SP, MU, SK, and GI (ANOVA, *p* > 0.05, Figure 5A–H). Therefore, ADM2 was removed in the subsequent experiments, and the remaining genes (β_2_GP1, CAMK1G, CIDE3, FAM213A, HSL, KRT222, NCEH1) were selected as the tissue-specific genes in AF.

### 3.5. Expression Characteristics of Adipose Tissue-Specific Genes under Starvation

**Figure 6:**
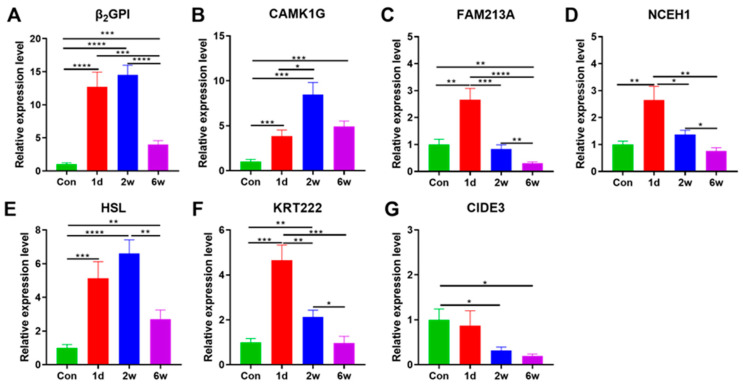
RT-PCR expression of tissue-specific genes at different starvation times in AF of *A. grunniens*. (**A**), β_2_GP1; (**B**), CAMK1G; (**C**), FAM213A; (**D**), NCEH1; (**E**), HSL; (**F**), KRT222; (**G**), CIDE3. Data were analyzed by Student’s *t*-test. * Refers to a significant difference between starvation times (*, *p* < 0.05; **, *p* < 0.01; ***, *p* < 0.001; ****. *p* < 0.0001), results were indicated as mean ± SEM; *n* = 9.

To further validate the role of these tissue-specific genes, we next conducted starvation stress to block food intake and improve the depletion of deposited fats in AF tissue. Results show that, compared with the control, β_2_GP1 was upregulated dramatically during starvation (T, *p* < 0.001). Similarly, CAMK1G at 2 w was distinctly higher than the control, 6 w (T, *p* < 0.001), and 1 d (T, *p* = 0.019). The transcriptional expression of FAM213A and NCEH1 at 1 d was significantly higher than the control (T, *p* < 0.01), of which FAM213A was extremely significantly downregulated at 2 w and 6 w (T, *p* < 0.01), Likewise, NCEH1 was significantly reduced at 2 w (T, *p* = 0.017) and 6 w (T, *p* < 0.01). HSL at 1 d and 2 w were significantly different from the control and 6 w (T, *p* < 0.01). The expression of KRT222 was increased at 1d of starvation compared to other times (T, *p* < 0.01). CIDE3 reduced as starvation duration increased, and the difference was significant only after 2 w (T, *p* = 0.017) and 6 w (T, *p* = 0.045, Figure 6).

### 3.6. Expression Characteristics of Adipose Tissue-Specific Genes under Hypothermia

**Figure 7:**
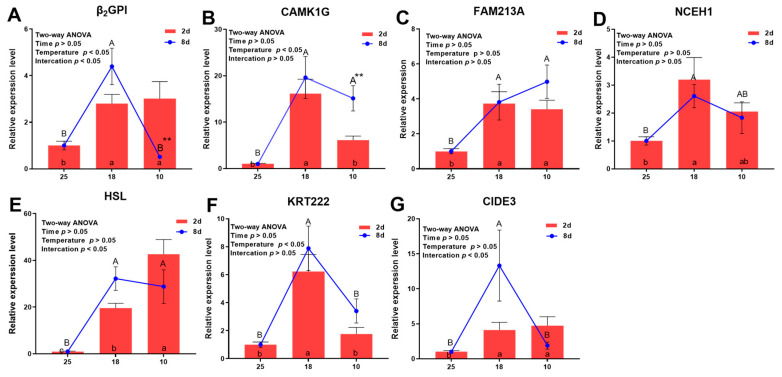
RT-PCR expression of tissue-specific genes at different temperatures and times in AF of *A. grunniens*. (**A**), β_2_GP1; (**B**), CAMK1G; (**C**), CIDE3; (**D**), FAM213A; (**E**), HSL; (**F**), KRT222; (**G**), NCEH1. Data were analyzed by one-way ANOVA, two-way ANOVA and *t*-test. Different letters indicate significant differences between the control group and the experimental group (*p* < 0.05), lowercase letters represent significant differences at 2 d and uppercase letters represent significant differences at 8 d. Asterisk Refers to a significant difference between different times at the same temperature level (** *p* < 0.01), results were indicated as mean ± SEM; *n* = 9.

The key role played by tissue-specific genes was further validated under hypothermic stress in order to comprehend how hypothermia stimulates lipolysis and thermogenesis, as shown in Figure 7. At LT-18, there was no significant difference in gene expression at different times (T, *p* > 0.05). At LT-10, only β_2_GP1 significantly decreased with time increases (T, *p* < 0.01), whereas CAMK1G dramatically increased (T, *p* < 0.01). At 2 d, all genes were markedly increased at LT-18 (ANOVA, *p* < 0.05). As the temperature decreased, the expressions of CAMK1G, NCEH1, and KRT222 were not dramatically different from the control (ANOVA, *p* > 0.05). At 8 d, all genes were significantly increased at LT-18 (ANOVA, *p* < 0.05) but β_2_GP1, NCEH1, KRT222, and CIDE3 returned to their original levels at LT-10 (ANOVA, *p* > 0.05). Interaction analysis showed that β_2_GP1, HSL, and CIDE3 were affected by the interaction between time and temperature (ANOVA, *p* < 0.05).

### 3.7. Comprehensive Analysis of Tissue-Specific Genes under Hypothermia and Starvation

**Figure 8:**
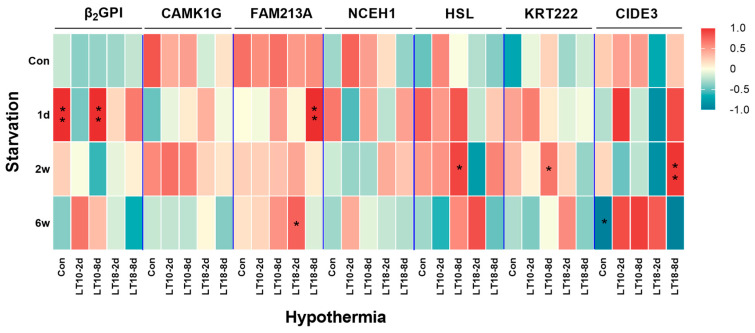
Correlation analysis heatmap of adipose tissue−specific genes under starvation and hypothermia. Data were analyzed by Pearson with SPSS 26.0. * Represents the statistical difference (*, *p* < 0.05; **, *p* < 0.01). The results are presented as mean ± SEM; *n* = 9.

Based on the aforementioned information, the important role of tissue-specific genes in exogenous stress was comprehensively analyzed. The results of the Pearson correlation analysis are shown in Figure 8. We discovered that starvation stress and hypothermic stress mainly affected the expression of β_2_GP1, FAM213A, HSL, KRT222, and CIDE3 genes, while there was no significant correlation increase in CAMK1G and NCFH1. At 2 d of short-term hypothermia, only FAM213A was significantly correlated at 6 w of starvation. At 8 d of prolonged hypothermia, starvation for 1 d resulted in increases in β_2_GP1 and FAM213A, and starvation for 2 w resulted in increases in HSL, KRT222, and CIDE3.

## 4. Discussion

Suitable internal reference genes could improve the reliability of RT-PCR results, thereby confirming the expression and function of specific genes. In summary, there is no common internal reference gene available for gene expression standardization under all conditions [34,35]. It is generally known that β-actin and GAPDH are the most used reference genes. However, it was found that β-actin and GAPDH exhibit remarkable differences in different tissues and experimental conditions [36,37,38]. Similarly, it was demonstrated in the present study that GAPDH and β-actin are not applicable as reference genes for adipose tissue in freshwater drum, which may be related to their functions. β-actin is an essential component of cell structure maintenance, cell motility, cytoplasmic division, endocytosis, and cell adhesion [39], while it has been found that β-actin is differentially expressed at different stages of preadipocyte differentiation [40]. GAPDH plays a vital role in glycolytic processes [41] and has also been shown to be unsuitable as a common reference gene in adipose tissue due to its differential expression levels in brown adipose tissue (BAT) and white adipose tissue (WAT) [42]. In addition to the above-mentioned classical internal reference genes, many studies have also found that RPL7 [43,44] and Rpl13α [45,46] can also be used as internal references. In the present study, 60SRP was the most stably expressed gene in freshwater drum adipose tissue. As a member of the ribosomal protein family, 60SRP plays an essential role in protein synthesis, cell proliferation, apoptosis, multiple regulations of development, and malignant transformation. It has been shown that 60SRP can serve as an internal reference gene for fish tissues [47], which has the same results as the present study.

Tissue-specific genes are a kind of gene that are specifically expressed in certain tissues. Previous studies have found that these adipose tissue-specific genes have various functions, such as lipid transport (β_2_GP1) [48], fat synthesis (CIDE3) [49], lipolysis (HSL, NCEH1) [50,51,52,53], calcium metabolism, cell proliferation and apoptosis (CAMK1G) [54,55], protective cells (KRT222) [56,57] and antioxidant (FAM213A) [58,59]. Identifying the specificity of adipose genes is important for understanding the development and regulation of adipose tissue.

As an endocrine organ, adipose tissue is critical for a variety of physiological functions such as postprandial uptake of glucose and fatty acids, appetite control, and insulin sensitivity [60]. Dysfunction of adipose tissue will induce metabolic dysregulation, which in turn inhibits substrate uptake, secretory profile alteration, angiogenesis stimulation, and inflammatory cell recruitment [60]. Therefore, maintaining a relatively stable state and function of adipose tissue is vital for the health of animals. Next, we further evaluate the function of the adipose tissue-specific genes with adverse stress that could impair the function of adipose tissue. As prevalent extrinsic variables, starvation and hypothermia have a huge impact on lipid metabolism. Studies have shown that both hypothermia and starvation lead to impaired triglyceride metabolic processes [61,62]. Hypothermic stress was found to have a facilitative effect on lipid metabolism in species such as *C. carpio* [63], *Sparus aurata* [64], *D. rerio* [15]. Starvation stress was also found to consume body fat in species such as *Pseudobagrus vachelli* [65], *Anguilla anguilla* [66], *Gadus morhua* and *Esox lucius* [67]. Similarly, we found that CIDE3 levels were decreasing under prolonged LT-10 as well as starvation, indicating that starvation stress and prolonged cold stimulation cause a decrease in the rate of lipid synthesis and accumulation. The significant increase in apolipoprotein β_2_GP1, lipolytic genes HSL and NCEH1 under LT-18, and starvation indicated that the process of lipolytic translocation was accelerated, and the organism also promoted cell proliferation and differentiation by increasing the level of CAMK1G. These results suggest that promoting lipolytic metabolism is one of the most important ways to ensure energy supply in fish. In addition, fish organism cells transmit the hypothermia signal to the nucleus through various stress pathways, initiating the hypothermic stress response, and establishing new intracellular homeostasis after sensing a hypothermia stimulus [68]. For instance, the fish central nervous system is stimulated by hypothermia to activate ion channels as hypothermia receptors during hypothermic stress, which excites the inward flow of extracellular calcium ions and related kinases to implement the transmission of hypothermia signals [68]. Since CAMK1G can release calcium ions, this may also be one of the reasons for its increase at LT-18 and decrease at LT-10. Extensive studies showed that changes in environmental conditions could disrupt the antioxidant defense system in aquatic animals, resulting in stress responses [69,70,71]. In response to detrimental external environments, we found that freshwater drum protected the organism from oxidative stress by increasing the expression of FAM213A and KRT222. The above results indicate that both hypothermia and starvation may lead to molecular, cellular, and tissue damage. However, a distinct result showed that the expression of nearly all genes was reduced during prolonged starvation and hypothermia, indicating that the regulatory ability of these genes in fish is not unlimited. If the stimulus exceeds a certain intensity, it will cause structural damage to tissues or cells, reducing the fish’s ability to self-regulate. However, lipid transport β_2_GP1 and lipolysis HSL genes are still increased during prolonged starvation compared to the control, which indicates that fish will reduce their metabolic level to conserve energy. Furthermore, fish continue to keep their metabolism above a certain level as much as possible to ensure normal life activities [72]. Additionally, through correlation analysis of starvation and hypothermia, we found a remarkable positive correlation between starvation and prolonged hypothermia. The above results demonstrate that in the early stages of starvation and short-term hypothermia, the body’s energy requirements are relatively low, and energy metabolism and resistance to external environmental changes may be altered by increasing β_2_GP1 and FAM213A. As starvation prolongs and temperature decreases, the body regulates its metabolic rate to supply life-sustaining caloric energy and protect the body’s health, mainly by altering the expression of FAM213A, HSL, KRT222, and CIDE3.

The above findings indicate the sensitivity of freshwater drums to environmental influences. When they are under stress, their normal metabolic processes may not provide sufficient energy, therefore mobilizing adipose tissue to take part in the regulation of energy homeostasis. The adipose-specific genes identified in this paper (β_2_GP1, CAMK1G, CIDE3, HSL, NCEH1, KRT222, and FAM213A) may play different essential roles in protecting the organism from exogenous stress and in promoting lipolysis to provide energy to the organism, and it is speculated that these genes may be key regulators of adipose tissue in response to exogenous stress states.

## 5. Conclusions

In this study, we identified a stable internal reference gene (60SRP) in adipose tissue and validated adipose tissue-specific genes by using 60SRP. We also verified the important functions of these specific genes under starvation stress and hypothermic stress. For a variety of roles, β_2_GP1 in lipid transport; CAMK1G in adipocyte proliferation and differentiation; CIDE3 in lipid synthesis; HSL, NCEH1 in lipolysis for energy supply; KRT222, FAM213A in protecting adipose cell structure against exogenous stress. These results provide tissue-specific targets for the mechanisms of lipid regulation in freshwater drums under exogenous stress, which could contribute to the development of freshwater drum culture.

## Figures and Tables

**Table 1:** Primer sequence of real-time fluorescence quantitative RT-PCR.

GenBank	Gene	Primer Sequence (5′—3′)	Amplification Efficiency	R^2^
XP_008328442.1	*β-actin*	F: AGGCTGTGCTGTCCCTGTAT R: GCTGTGGTGGTGAAGGAGTAG	102.08	0.9995
AEI83278.1	*B2M*	F: CCTGGAAAGTTCGGCAGTAG R: TCCACGTTCTTGGTCAGATG	98.11	0.9997
KKF31758.1	*EF1α*	F: TGACAACTTCAACGCTCAGG R: ATGGGCTTCTGTGGAATGAG	102.26	0.9984
AAL31950.1	*18S*	F: TCAGCGTGTGTCTACCCTTC R: CCTCACTAAACCATCCAATCG	106.95	0.9990
XP_018517365.1	*60SRP*	F: CAAAGGACATCAAGGCCATC R: GAGCCACTACAGCACCACCT	106.80	0.9951
CAG01324.1	*EEF1B*	F: GATGAGGGTGGGCTTCTTG R: ATGTTGACCTGTTCGGCTCT	107.28	0.9998
XP_010741722.1	*GAPDH*	F: ATGACCCTTTCATCGACCTG R: GCTTCACCCCATTTGATGATT	104.21	0.9990
XP_010732148.1	*RPl7*	F: GATGCTGGCTGAGAAGAAGG R: GCCGTTGATACCTCTGATCC	98.11	0.9987
XP_010747631.1	*RPS4*	F: GACAAGCTGACCGGAGTGTT R: CCAGCAGGGTAGGTGATGTC	95.76	0.978
XP_010738284.2	*SEC62*	F: GCCATCACTTCTGGTTCCTC R: CCATCCTTCTTTTCGCTGTC	103.98	0.9998
XP_005808493.1	*ufm1*	F: GCCGTTCACAGCAGTTTG R: GTCTCCTTGTCCTCCCACTCT	107.86	0.9987
XP_010742647.2	*β_2_GP1*	F: GGCAGTATCCTCACCCCATC R: CCTTCTGAGGTCCATCCAGC	99.17	0.9988
KKF21127.1	*CAMK1G*	F: TACATGCTCGGCTCCACTCT R: TCTCCTTCACGCTCAACTCG	108.54	0.9986
KKF23363.1	*KRT222*	F: GAGAGTGCAGAAGGTCACGG R: GGGGAGGCTGTCCTGTTTAG	94.77	0.9774
XP_010735223.1	*ADM2*	F: GCATGAAAGCAGCCTTGTCG R: CATGTTCCCAAGACGCAACC	107.28	0.9541
XP_018535573.1	*CIDE3*	F: ACCCCACATCCAAACAGCAT R: TTTTTGGCAGCGTAACAGCG	92.21	0.9628
XP_019122735.1	*HSL*	F: TTGCTGAGATGAGGGTGGA R: ACAGGCTGGTCTATGTTCC	107.33	0.9657
XP_010730495.2	*NCEH1*	F: TATTAACGGTGGCGTTCGCT R: AAAGAAGCCAGGTGCATCGT	110.28	0.9964
XP_010741055.2	*FAM213A*	F: CCCGTGAAAGAAAGATGG R: GTCCAATGACGAACACCC	107.57	0.9736

The mRNA sequences for each gene were obtained from freshwater drum transcriptome sequencing database. RT-PCR primers were designed using Primer Premier 5.0.

## Data Availability

The data presented in this study are available on request from the corresponding author. The data are not publicly available due to being involved in another unpublished project.

## Supplementary Materials

The following supporting information can be downloaded at: https://www.mdpi.com/article/10.3390/cells12091328/s1, Figure S1: RNA integrity number by RNA 6000 Nano LabChip for the samples; Figure S2: Standard curve of tissue-specific candidate genes in *A. grunniens*; Table S1: Bestkeeper Standard Deviation and Coefficient of Variation.

## Abbreviations

60SRP: 60S ribosomal protein; ADM2, adrenomedullin 2; β_2_GP1, beta-2-glycoprotein 1-like; CAMK1G, calcium/calmodulin-dependent protein kinase type 1G; CIDE3, cell death activator CIDE-3-like; FAM213A, redox-regulatory protein FAM213A-like; HSL, hormone-sensitive lipase; KRT222, keratin-like protein KRT222; NCEH1, neutral cholesterol ester hydrolase 1.

## References

[1] Weil C., Lefèvre F., Bugeon J. (2012). Characteristics and Metabolism of Different Adipose Tissues in Fish. Rev. Fish Biol. Fish..

[2] Liu D., Mai K., Ai Q. (2015). Tumor Necrosis Factor Alpha Is a Potent Regulator in Fish Adipose Tissue. Aquaculture.

[3] Fenzl A., Kiefer F.W. (2014). Brown adipose tissue and thermogenesis. Horm. Mol. Biol. Clin. Investig..

[4] Coelho M., Oliveira T., Fernandes R. (2013). Biochemistry of adipose tissue: An endocrine organ. Arch. Med. Sci..

[5] Kershaw E.E., Flier J.S. (2004). Adipose tissue as an endocrine organ. J. Clin. Endocrinol. Metab..

[6] Chatzifotis S., Panagiotidou M., Papaioannou N., Pavlidis M., Nengas I., Mylonas C.C. (2010). Effect of Dietary Lipid Levels on Growth, Feed Utilization, Body Composition and Serum Metabolites of Meagre (*Argyrosomus regius*) Juveniles. Aquaculture.

[7] Du Z.Y., Clouet P., Zheng W.H., Degrace P., Tian L.X., Liu Y.J. (2006). Biochemical Hepatic Alterations and Body Lipid Composition in the Herbivorous Grass Carp (*Ctenopharyngodon idella*) Fed High-Fat Diets. Br. J. Nutr..

[8] Dang H.Y. (2020). Effects of HSL gene on fat deposition in common *carp* and its regulatory mechanism. Shanghai Ocean Univ..

[9] Zeng B.H., Jin L.J., Wang Q., Su H.D. (2014). Pathology and prevention of liver injury in aquatic animals. Chongqing Aquac..

[10] Walks D., Lavau M., Presta E., Yang M.U., Björntorp P. (1983). Refeeding after Fasting in the Rat: Effects of Dietary-Induced Obesity on Energy Balance Regulation. Am. J. Clin. Nutr..

[11] Wilson R., Deasy W., Stathis C., Hayes A., Cooke M. (2018). Intermittent Fasting with or without Exercise Prevents Weight Gain and Improves Lipids in Diet-Induced Obese Mice. Nutrients.

[12] Bar N. (2014). Physiological and Hormonal Changes during Prolonged Starvation in Fish. Can. J. Fish. Aquat. Sci..

[13] van Dijk P.L.M., Hardewig I., HOlker F. (2005). Energy Reserves during Food Deprivation and Compensatory Growth in *Juvenile Roach*: The Importance of Season and Temperature. J. Fish Biol..

[14] Kooka K., Yamamura O. (2011). Winter Energy Allocation and Deficit of Juvenile Walleye Pollock *Theragra Chalcogramma* in the Doto Area, Northern Japan. Environ. Biol. Fishes.

[15] Lu D.L. (2019). Mechanisms of Fasting Enhance Cold Tolerance in Zebrafish (*Danio rerio*). Ph.D. Thesis.

[16] El-Sayed A.-F.M., Kawanna M. (2008). Optimum Water Temperature Boosts the Growth Performance of Nile Tilapia (*Oreochromis niloticus*) Fry Reared in a Recycling System. Aquac. Res..

[17] Snyder R.J., Hennessey T.M. (2003). Cold Tolerance and Homeoviscous Adaptation in Freshwater Alewives (*Alosa pseudoharengus*). Fish Physiol. Biochem..

[18] Song C., Liu B., Jiang S., Xiong Y., Sun C., Zhou Q., Jiang Z., Liu B., Zhang H. (2020). Anthraquinone Extract from Rheum Officinale Bail Improves Growth Performance and Toll–Relish Signaling-Regulated Immunity and Hyperthermia Tolerance in Freshwater Prawn *Macrobrachium nipponense*. 3 Biotech.

[19] Hsieh S.L., Kuo C.-M. (2005). Stearoyl–CoA Desaturase Expression and Fatty Acid Composition in Milkfish (*Chanos chanos*) and Grass Carp (*Ctenopharyngodon idella*) during Cold Acclimation. Comp. Biochem. Physiol. Part B Biochem. Mol. Biol..

[20] Hazel J.R. (1979). Influence of Thermal Acclimation on Membrane Lipid Composition of *Rainbow Trout* Liver. Am. J. Physiol. Regul. Integr. Comp. Physiol..

[21] Calder P.C. (2012). Mechanisms of Action of (n-3) Fatty Acids. J. Nutr..

[22] Spector A.A., Yorek M.A. (1985). Membrane Lipid Composition and Cellular Function. J. Lipid Res..

[23] Cao Y.C., Wang Z.X. (1991). Study on the mechanism of low temperature adaption in fish. Ⅱ.Effects of acclimation temperature on fitty acid composition and cholesterl content in mitochondrial membranes of muscle tissue of the *grass carp* and *mud carp*. J. South China Agric. Univ..

[24] Zhang X.D., Ye C.X., Xian J.A., Wang A.L. (2013). Research progress of fish cold stress. Feed. Ind..

[25] Liu W., Sun Z.Q., Xie H.W. (2016). The Progress of Gene Tissue Specificity Researches. Prog. Biochem. Biophys..

[26] Zhang Y.N. (2020). Screening on Pathways and Genes Related to Ovary Development of Oriental River Prawn (*Macrobrachium nipponense*) Based on Trancriptme and Metabolome. Master’s Thesis.

[27] Gao Y.F. (2022). Screening of Growth and Feeding Genes and Association Analysis between of wnt5b with Growth-Related Traits in *Bighead carp*. Master’s Thesis.

[28] Bo Y.Y. (2013). Exploited of Immune-Related Gene and Constructed of Microarray in the Common Carp. Master’s Thesis.

[29] Hernández-Gómez R.E., Contreras-Sánchez W.M., Hernández-Franyutti A., Perera-García M.A., Torres-Martínez A. (2021). Testicular Structure and Development of the Male Germinal Epithelium in the Freshwater Drum *Aplodinotus grunniens* (*Perciformes: Sciaenidae*) from the Usumacinta River, Southern Mexico. Acta Zool..

[30] Song C., Wen H., Liu G., Ma X., Lv G., Wu N., Chen J., Xue M., Li H., Xu P. (2022). Gut Microbes Reveal Pseudomonas Medicates Ingestion Preference via Protein Utilization and Cellular Homeostasis Under Feed Domestication in Freshwater Drum, *Aplodinotus grunniens*. Front. Microbiol..

[31] Chen J., Li H., Xu P., Tang Y., Su S., Liu G., Wu N., Xue M., Yu F., Feng W. (2022). Hypothermia-Mediated Apoptosis and Inflammation Contribute to Antioxidant and Immune Adaption in Freshwater Drum, Aplodinotus Grunniens. Antioxidants.

[32] Chen J., Song C., Wen H., Liu G., Wu N., Li H., Xue M., Xu P. (2022). miR-1/AMPK-Mediated Glucose and Lipid Metabolism under Chronic Hypothermia in the Liver of Freshwater Drum, Aplodinotus Grunniens. Metabolites.

[33] Wu N., Wen H., Xu P., Chen J., Xue M., Li J., Wang M., Song C., Li H. (2023). PPAR Signaling Maintains Metabolic Homeostasis under Hypothermia in Freshwater Drum (*Aplodinotus grunniens*). Metabolites.

[34] Peters I.R., Peeters D., Helps C.R., Day M.J. (2007). Development and Application of Multiple Internal Reference (Housekeeper) Gene Assays for Accurate Normalisation of Canine Gene Expression Studies. Vet. Immunol. Immunopathol..

[35] Mitter K., Kotoulas G., Magoulas A., Mulero V., Sepulcre P., Figueras A., Novoa B., Sarropoulou E. (2009). Corrigendum to “Evaluation of Candidate Reference Genes for QPCR during Ontogenesis and of Immune-Relevant Tissues of European Seabass (*Dicentrarchus labrax*)” [Comp. Biochem. Physiol. 153B (2009) 340–347]. Comp. Biochem. Physiol. Part B Biochem. Mol. Biol..

[36] Bustin S. (2000). Absolute Quantification of mRNA Using Real-Time Reverse Transcription Polymerase Chain Reaction Assays. J. Mol. Endocrinol..

[37] Nazari F., Parham A., Maleki A.F. (2015). GAPDH, β-Actin and Β2-Microglobulin, as Three Common Reference Genes, Are Not Reliable for Gene Expression Studies in Equine Adipose- and Marrow-Derived Mesenchymal Stem Cells. J. Anim. Sci. Technol..

[38] Glare E.M. (2002). Beta-Actin and GAPDH Housekeeping Gene Expression in Asthmatic Airways Is Variable and Not Suitable for Normalising mRNA Levels. Thorax.

[39] Xiao X., Li M., Wang K., Qin Q., Chen X. (2011). Characterization of Large Yellow Croaker (*Pseudosciaena crocea*) β-Actin Promoter Supports β-Actin Gene as an Internal Control for Gene Expression Modulation and Its Potential Application in Transgenic Studies in Fish. Fish Shellfish. Immunol..

[40] Ruan W., Lai M. (2007). Actin, a Reliable Marker of Internal Control?. Clin. Chim. Acta.

[41] Tossounian M.A., Zhang B., Gout I. (2020). The writers, readers, and erasers in redox regulation of GAPDH. Antioxidants.

[42] Zhang W.-X., Fan J., Ma J., Rao Y.-S., Zhang L., Yan Y.-E. (2016). Selection of Suitable Reference Genes for Quantitative Real-Time PCR Normalization in Three Types of Rat Adipose Tissue. Int. J. Mol. Sci..

[43] Zhang Z., Hu J. (2006). Development and Validation of Endogenous Reference Genes for Expression Profiling of Medaka (*Oryzias latipes*) Exposed to Endocrine Disrupting Chemicals by Quantitative Real-Time RT-PCR. Toxicol. Sci..

[44] Liu Y., Cheng J., Fan K., Xia Y., Zhang Z., Liu Y., Liu P. (2021). Evaluation of Potential Reference Genes by Quantitative RT-qPCR Analysis of *Takifugu rubripes* under Normal Conditions and after Cryptocaryon Irritans Infection. Aquac. Res..

[45] Rassier G.T., Silveira T.L.R., Remião M.H., Daneluz L.O., Martins A.W.S., Dellagostin E.N., Ortiz H.G., Domingues W.B., Komninou E.R., Kütter M.T. (2020). Evaluation of qPCR Reference Genes in GH-Overexpressing Transgenic Zebrafish (*Danio rerio*). Sci. Rep..

[46] Xu H., Li C., Zeng Q., Agrawal I., Zhu X., Gong Z. (2016). Genome-Wide Identification of Suitable Zebrafish *Danio Rerio* Reference Genes for Normalization of Gene Expression Data by RT-qPCR. J. Fish Biol..

[47] Rojas-Hernandez N., Véliz D., Vega-Retter C. (2019). Selection of Suitable Reference Genes for Gene Expression Analysis in Gills and Liver of Fish under Field Pollution Conditions. Sci. Rep..

[48] Bettacchioli E., Nafai S., Renaudineau Y. (2019). News and Meta-Analysis Regarding Anti-Beta 2 Glycoprotein I Antibodies and Their Determination. Clin. Immunol..

[49] Puri V., Konda S., Ranjit S., Aouadi M., Chawla A., Chouinard M., Chakladar A., Czech M.P. (2007). Fat-Specific Protein 27, a Novel Lipid Droplet Protein That Enhances Triglyceride Storage. J. Biol. Chem..

[50] Lan Y.-L., Lou J.-C., Lyu W., Zhang B. (2019). Update on the Synergistic Effect of HSL and Insulin in the Treatment of Metabolic Disorders. Ther. Adv. Endocrinol. Metab..

[51] Lampidonis A.D., Rogdakis E., Voutsinas G.E., Stravopodis D.J. (2011). The Resurgence of Hormone-Sensitive Lipase (HSL) in Mammalian Lipolysis. Gene.

[52] Sekiya M., Yamamuro D., Ohshiro T., Honda A., Takahashi M., Kumagai M., Sakai K., Nagashima S., Tomoda H., Igarashi M. (2014). Absence of Nceh1 Augments 25-Hydroxycholesterol-Induced ER Stress and Apoptosis in Macrophages. J. Lipid Res..

[53] Igarashi M., Osuga J., Isshiki M., Sekiya M., Okazaki H., Takase S., Takanashi M., Ohta K., Kumagai M., Nishi M. (2010). Targeting of Neutral Cholesterol Ester Hydrolase to the Endoplasmic Reticulum via Its N-Terminal Sequence. J. Lipid Res..

[54] Takemoto-Kimura S., Ishihara-Ageta N., Nonaka M., Okuno H., Bito H. (2007). Regulation of Dendritogenesis via a Lipid Raft-Associated Ca2+/Calmodulin-Dependent Protein Kinase CLICK-III/CaMKIγ. Neurosci. Res..

[55] Li Z., Tian Y., Wang L., Li Z., Chen S., Li L., Liu Y., Li W., Pang Z., Ma W. (2022). Comparative Transcriptomics Analyses and Revealing Candidate Networks and Genes Involved in Lordosis of the Yunlong Grouper (*Epinephelus Moara* ♀ × *Epinephelus Lanceolatus* ♂). Aquaculture.

[56] Herrmann H., Hesse M., Reichenzeller M., Aebi U., Magin T.M. (2003). Functional complexity of intermediate filament cytoskeletons: From structure to assembly to gene ablation. Int. Rev. Cytol..

[57] Kirfel J., Magin T.M., Reichelt J. (2003). Keratins: A Structural Scaffold with Emerging Functions. Cell. Mol. Life Sci..

[58] Oh C.K., Ha M., Han M.E., Heo H.J., Myung K., Lee Y., Oh S.O., Kim Y.H. (2020). FAM213A is linked to prognostic significance in acute myeloid leukemia through regulation of oxidative stress and myelopoiesis. Hematol Oncol..

[59] Chen Y.-F., Wei Y.-Y., Yang C.-C., Liu C.-J., Yeh L.-Y., Chou C.-H., Chang K.-W., Lin S.-C. (2019). miR-125b Suppresses Oral Oncogenicity by Targeting the Anti-Oxidative Gene PRXL2A. Redox Biol..

[60] Matafome P., Seiça R. (2017). Function and Dysfunction of Adipose Tissue. Adv. Neurobiol..

[61] Cai R.J., Zhang J., Huang J.S., Shi G., Pan C.H., Xie R.T., Chen G., Zhang J.D., Wang Z.L., Tang B.G. (2021). Effects of low temperature stress on the expression of genes related to lipid metabolism of juvenile cobia, Rachycentron canadum. Haiyang Xuebao.

[62] Xu C. (2018). Study on the Triglycerol Catabolism, Dietary Lipid and α-Lipoic acid on Chinese Mitten Crab Eriocheir Sinensis. Ph.D. Thesis.

[63] Dietrich M.A., Hliwa P., Adamek M., Steinhagen D., Karol H., Ciereszko A. (2018). Acclimation to Cold and Warm Temperatures Is Associated with Differential Expression of Male *Carp* Blood Proteins Involved in Acute Phase and Stress Responses, and Lipid Metabolism. Fish Shellfish. Immunol..

[64] Mateus A.P., Costa R., Gisbert E., Pinto P.I.S., Andree K.B., Estévez A., Power D.M. (2017). Thermal Imprinting Modifies Bone Homeostasis in Cold Challenged Sea Bream (*Sparus aurata* L.). J. Exp. Biol..

[65] Qin C.J., Shao T., Yang J.P., Gong Q., Li L.J. (2015). The effect of starvation on lipid metabolism of darkbarbel catfish, *Pelteobagrus vachelli*. Acta Hydrobiol. Sin..

[66] Larsson A., Lewander K. (1973). Metabolic Effects of Starvation in the *Eel*, *Anguilla anguilla* L. Comp. Biochem. Physiol. Part A Physiol..

[67] Ince B.W., Thorpe A. (1976). The Effects of Starvation and Force-Feeding on the Metabolism of the Northern Pike, *Esox lucius* L. J. Fish Biol..

[68] Long Y., Ge G.D., Li X.X., Cui Z.B. (2021). Regulatory mechanisms of low-temperature stress response in fish. Acta Hydrobiol. Sin..

[69] Zhao C.Y., Zhou X., Bing X.W., Wang G.Q. (2010). Effects of starvation on digestive enzyme activities and some immune indexes in broodstock red swamp crawfish *Procambarus clarkii*. J. Dalian Fish Univ..

[70] Li D.P., Liu S.Y., Xie C.X., Zhang X.Z. (2008). Effects of water temperature on serum content of reactive oxygen species and antiox idant defense system in Chinese sturgeon, *Acipenser sinensis*. Acta Hydrobiol. Sin..

[71] Chen H.G., Ma S.W., Lin Q., Gan J.L., Cai W.G., Jia X.P. (2009). Effects of tribu tyltin chloride (TBTCl) on SOD activities, MDA contents and GPx activities in gill and liver of the black porgy (*Sparus macro cephalus*). South China Fish. Sci..

[72] Du Z.Y., Liu Y.J., Tian L.X., Cao J.M., Liang G.Y., He J.G. (2003). Effects of starvation on visceral weight and main biochemical compsition of the muscle, liver and serum in the Janpanese sea bass (*Lateolabrax japonicus*). Acta Zool. Sin..

