# Supplementary material for: Validation and Functional Analysis of Reference and Tissue-Specific Genes in Adipose Tissue of Freshwater Drum, Aplodinotus grunniens, under Starvation and Hypothermia Stress
Source: Cells. 2023 May 6;12(9):1328. doi: 10.3390/cells12091328 (PMC10177597; doi:10.3390/cells12091328)
Supplement: Supplementary file 1 [file cells-12-01328-s001.zip › cells-2183105-supplementary.pdf]

Validation and Functional Analysis of Reference and Tissue-Specific Genes in Adipose Tissue of Freshwater Drum, *Aplodinotus grunniens* under Starvation and Hypothermia Stress

Miaomiao Xue[1], Haibo Wen [1,2], Pao Xu [1,2], Jianxiang Chen [1], Qingyong Wang [1], Yongkai Tang [1,2], Xueyan Ma [1,2], Guohua Lv [1,2], Changyou Song [1,2,*], Hongxia Li [1,2,*]

Supplementary Materials:

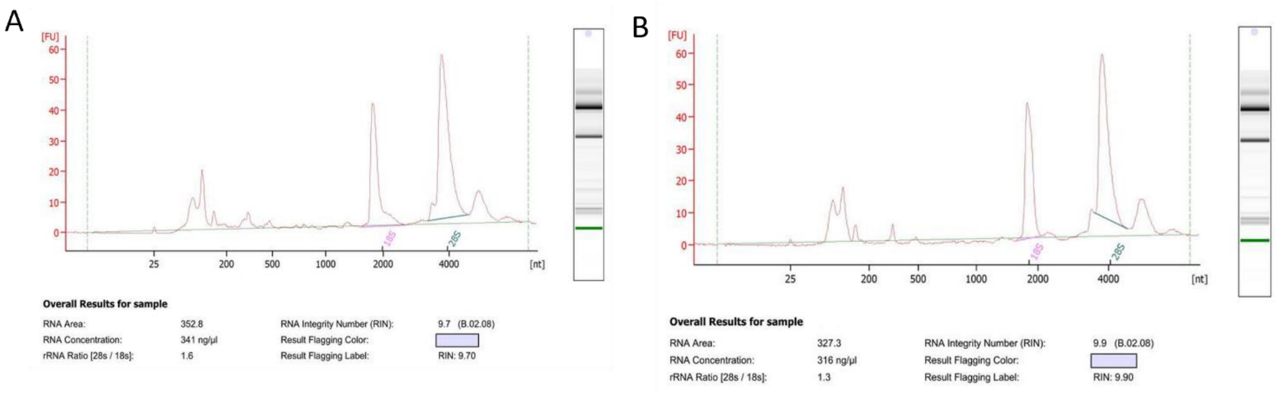

Figure S1. RNA integrity number by RNA 6000 Nano LabChip for the samples.

The concentration degree of each peak in the figure represents the integrity of RNA in the sample. The peak concentration represents a low degradation rate of RNA in the sample. The RIN value represents the integrity of the RNA sample, with higher values indicating better RNA integrity.

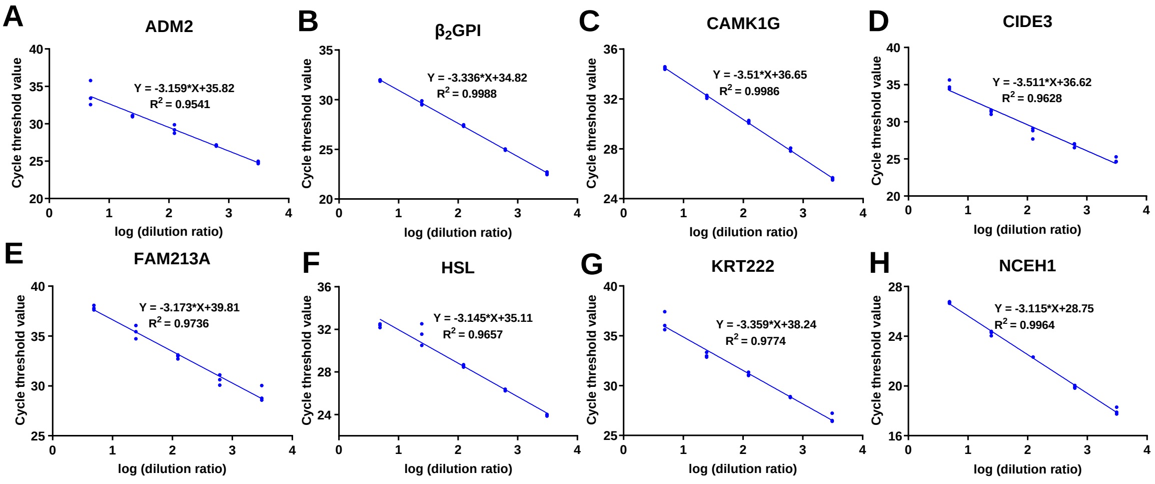

Figure S2. Standard curve of tissue-specific candidate genes in *A. grunniens*.

(**A**), adrenomedullin 2, ADM2; (**B**), beta-2-glycoprotein 1-like, β_2GP1; (**C**), calcium/calmodulin-dependent protein kinase type 1G, CAMK1G; (**D**), cell death activator CIDE-3-like, CIDE3; (**E**), redox-regulatory protein FAM213A-like, FAM213A; (**F**), hormone-sensitive lipase, HSL; (**G**), keratin-like protein KRT222,

KRT222; (**H**), neutral cholesterol ester hydrolase 1, NCEH1.

Table S1. Bestkeeper Standard Deviation and coefficient of variation

Reference gene	RPS4	β-actin	RPl7	60SRP	EF1α	ufm1	B2M	EEF1B	SEC62	18S	GAPDH
SD	0.91	0.93	1.04	1.14	1.37	1.79	1.81	1.85	2.15	3.46	3.80
CV	4.36	4.70	5.46	6.20	6.75	6.31	9.22	8.42	7.45	14.52	15.88
